# Wearable sensors for telehealth based on emerging materials and nanoarchitectonics

**DOI:** 10.1038/s41528-023-00261-4

**Published:** 2023-06-02

**Authors:** Jayraj V. Vaghasiya, Carmen C. Mayorga-Martinez, Martin Pumera

**Affiliations:** 1 Center for Advanced Functional Nanorobots, Department of Inorganic Chemistry, Faculty of Chemical Technology, University of Chemistry and Technology Prague, Technická 5, 166 28, Prague, Czech Republic; 2 Faculty of Electrical Engineering and Computer Science, VSB - Technical University of Ostrava, 17. listopadu 2172/15, 70800 Ostrava, Czech Republic

**Keywords:** Materials for devices, Two-dimensional materials

## Abstract

Wearable sensors have made significant progress in sensing physiological and biochemical markers for telehealth. By monitoring vital signs like body temperature, arterial oxygen saturation, and breath rate, wearable sensors provide enormous potential for the early detection of diseases. In recent years, significant advancements have been achieved in the development of wearable sensors based on two-dimensional (2D) materials with flexibility, excellent mechanical stability, high sensitivity, and accuracy introducing a new approach to remote and real-time health monitoring. In this review, we outline 2D materials-based wearable sensors and biosensors for a remote health monitoring system. The review focused on five types of wearable sensors, which were classified according to their sensing mechanism, such as pressure, strain, electrochemical, optoelectronic, and temperature sensors. 2D material capabilities and their impact on the performance and operation of the wearable sensor are outlined. The fundamental sensing principles and mechanism of wearable sensors, as well as their applications are explored. This review concludes by discussing the remaining obstacles and future opportunities for this emerging telehealth field. We hope that this report will be useful to individuals who want to design new wearable sensors based on 2D materials and it will generate new ideas.

## Introduction

Rapid expansion in the development and use of wearable sensors with a compound annual growth rate (CAGR) of 18.3% from 327.6 million in 2021 to 1,487 million 2030^1^, is mainly responsible for the rising demand in telehealth and real-time health monitoring. As the foundation of the human-machine interface, a wide range of wearable sensors have been established for monitoring biological (*i.e*., sweat biomarkers such as cortisol, glucose, pH, etc.), mechanical (*i.e*., pressure, strain, and stress), temperature, and other input signals^2–4^. In recent years, this field has made considerable advancements due to the development of sensing materials and flexible electronics with wireless data transfer systems, which could endorse and pave the way for a new era of digital healthcare and medicine. A remote health monitoring system (RHMS), also known as telehealth, would be useful for all patients suffering from serious illnesses such as diabetes, cardiovascular disease, mental illness, cancer, and hypertension, and for COVID-19 infections to be monitored in real-time and remotely^5^. RHMS provides the ability to continually gather information on health conditions such as physiological parameters, metabolism status, and body movements. These real-time data recordings allow for close monitoring of health conditions and prompt action recommendations if needed. Typically, an RHMS comprises three components: a sensing device, a data transfer device, and a power supply device^6^. The sensor device senses real-time bio-signals and transforms them into electrical impulses that are wirelessly relayed to the cloud or mobile phone for further analysis. Both sensing and data transmission processes necessitate the use of a power source. In comparison to other electronics, the RHMS has unique power source requirements such as flexibility, stretchability, biocompatibility, wearability, and comfort^7–9^. Therefore, both sensing and power supply components are crucial to an RHMS.

The successful use of graphene in numerous biosensing devices has prompted research into other materials that are similar to graphenes such as transition metal carbides (MXenes), metal phosphorus chalcogenide, transition metal dichalcogenides (TMDs), hexagonal boron nitride (h-BN), and black phosphorous (BP)^10–12^. These 2D layered materials are highly suitable for developing high-performance skin-integrated wearable sensors because they have a range of compositions, structures, and functionality^13–15^. 2D materials are potential candidates for sensing various bio-signals, movements, and electrocardiograms due to their outstanding electro-mechanical characteristics. Wearable sensors and biosensors made of 2D materials are now capable of more than just sensing ordinary physical signals, such as heartbeat^16^, body temperature^17^, muscle fatigue^18^, and vocal cord vibration^19^ as they incorporate more specific biomarkers that may be identified in a patient with lung, liver, pancreatic, or breast cancer^20,21^. Additionally, these 2D materials-based wearable sensors provide real-time health status data that can be recorded by cloud or mobile devices, opening up new possibilities for personalized digital medicine.

The flexibility, breathability, and stretchability of wearable sensors are compatible with human skin and offer comfort and compliance with body movements^22,23^. In recent years, wearable technology has grown significantly and many 2D materials are already used as skin-adhesive wearable sensors^24^. Our knowledge of how wearable devices can significantly influence personalized health monitoring has just begun. Nowadays, many research reports are being published related to various facets of this extremely important subject, which is encouraging many new researchers. In this review, we highlighted the usage of 2D materials in wearable sensing technologies for real time and remote health monitoring that in our best understanding was not reported before. Additionally, the purpose of this review is to enlighten both new researchers and the scientific community about recent achievements and challenges in wearable sensors and telehealth.

**Fig. 1:**
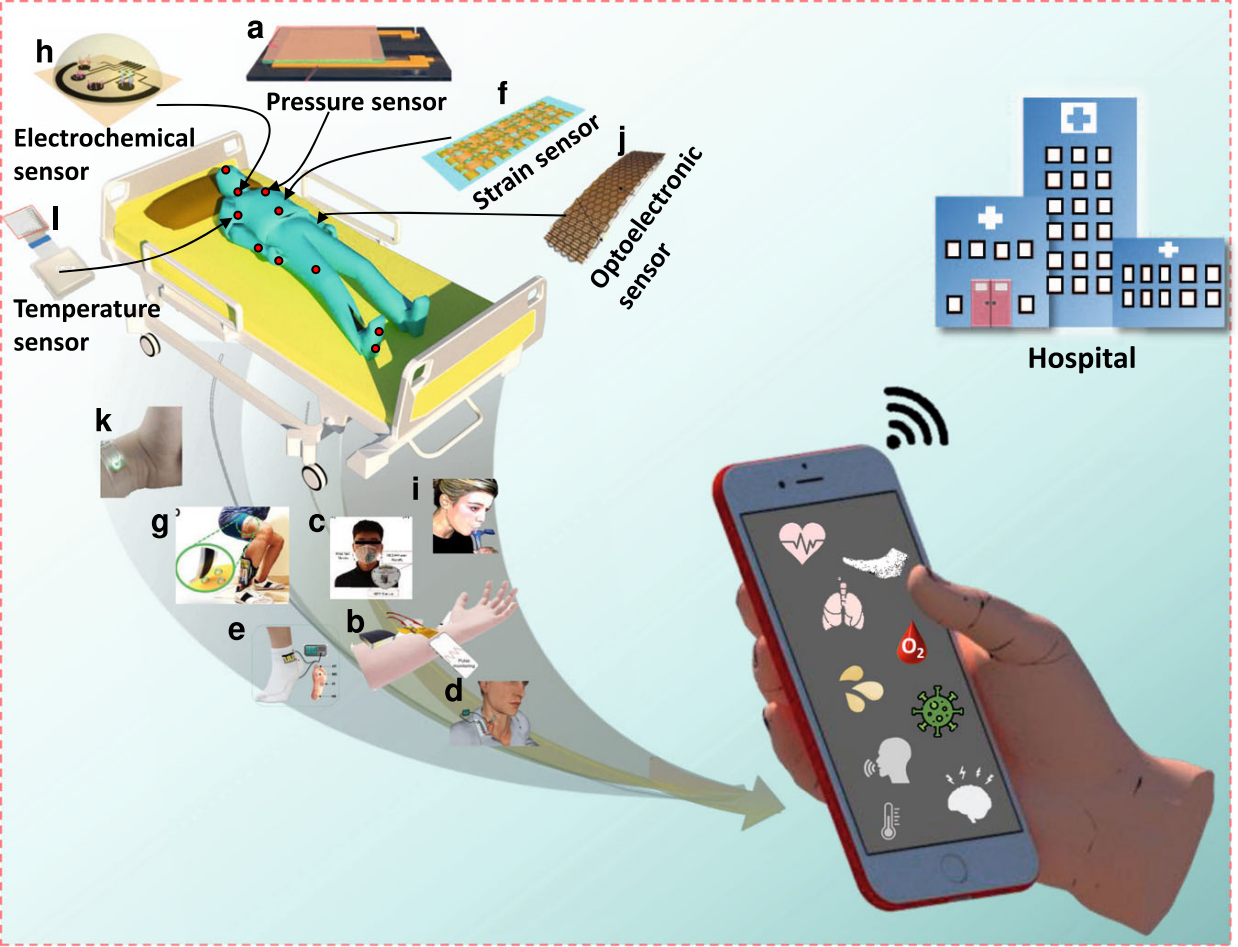
Overview of this review. Recently developed wearable sensors based on 2D materials for remote health monitoring; **a**–**e** Wearable pressure sensor for monitoring wrist (radial artery) pulse, respiratory cycle, neck (carotid artery) pulse, and body movements. Reprinted with permission^42,45^_._ Copyright 2021, American Chemical Society_,_ and Copyright 2020, Wiley. **f**, **g** Wearable strain sensor for muscle fatigue monitoring. Reprinted with permission^18,51^_._ Copyright 2020, Elsevier and Copyright 2021, Wiley. **h**, **i** Wearable electrochemical sensor for identifying biomarkers, Reprinted with permission^59^_._ Copyright 2020, Elsevier. **j**, **k** Wearable optoelectronic sensor for arterial oxygen saturation monitoring. Reprinted with permission^56^_._ Copyright 2019, The Authors, some rights reserved; exclusive licensee American Association for the Advancement of Science. Distributed under a Creative Commons Attribution-NonCommercial License 4.0 (CC BY-NC) http://creativecommons.org/licenses/by-nc/4.0/. **l** Wearable temperature sensor for body temperature monitoring. Reprinted with permission^17^_._ Copyright 2022, The Authors, some rights reserved; exclusive licensee American Association for the Advancement of Science. Distributed under a Creative Commons Attribution-NonCommercial License 4.0 (CC BY-NC) http://creativecommons.org/licenses/by-nc/4.0/.

Herein, we review the current state of the most well-known 2D materials-based wearable sensors employed for RHMS (Fig. 1). In particular, we highlight the distinctive features of 2D materials that make them special and desirable in the wearable sensor devices, with a description of different wearable sensors for real-time and remotely monitor physiological parameters. An overview of sensing mechanisms, including piezoelectric, resistive, capacitive, optical, and electrochemical systems, is discussed. Furthermore, it comprehensively reviews each kind of wearable sensor (pressure, strain, electrochemical, temperature, and optoelectronic sensors) and demonstrates how they can be used for everyday activity tracking and remote health monitoring. In addition, existing challenges and opportunities of wearable sensors in telehealth are highlighted. The future outlooks of wearable sensors and telehealth are briefly discussed with possible concrete instances, which will help advance scientific research and wearable sensor development for personalized healthcare.

## Why nanoarchitectonics 2D materials for telehealth?

With remarkable advancements in nanoscience and nanotechnology, many types of nanomaterials have been synthesized and employed in wearable sensors^25^. Among them, a type of 2D layered nanoarchitectonics materials have recently gained a lot of attention. 2D materials represent a new class of nanomaterials with excellent physicochemical properties (*e.g*., high transparency, large surface area, flexibility, high selectivity, and stretchability), enhanced electrochemical and optical properties, and in many cases are biocompatible^26^. 2D materials families consist of graphene, MXenes, pnictogens, TMDs, h-BN, and metal thiophosphates (MPS_3_)^26–28^. Research into graphene and rGO has become increasingly successful over the past ten years. Further, scientists have broadened their investigation into 2D materials to include Ti_3_C_2_T_x_, h-BN, MoS_2_, WSe_2_, FePS_3_, BP, and so on^28,29^. These materials have received a lot of attention in sensing and detection techniques. In this field, 2D materials have many benefits. For example, the conductivity of 2D materials can be tuned by doping or functionalization with polymer materials, adding layers, and managing structural detects^28^. In general, 2D materials are monolayers with few nanometers of thickness, which provide a large surface area to volume ratio and many reactive sites between the analytes and material^27,30^. Excellent mechanical stability and flexibility of 2D materials can be used with cutting-edge technologies such as flexible and wearable electronics. Specifically, 2D layered materials such as MXenes, graphene, and their derivatives are progressively taking over in the field of wearable sensors due to their superior conductivity, hydrophilicity, electrocatalytic activity, easy-to-tune morphology, and excellent mechanical properties^31^. By taking into account the benefits of these materials and the target needs of devices, a new sensing system is developed by merging either MXenes or graphene with other appropriate materials (i.e. polymers, metal oxides, nanoparticles, etc.), which can enhance the synergistic effect between both materials and provide an excellent performance sensor with broad response range and high sensitivity. Also, TMDs have considerable potential for real-time biomarker identification^32^. Remarkable properties of TMDs such as excellent carrier mobility, optical properties, tuneable band edge, mechanical flexibility, and planar structure, make them promising materials for the creation of health monitoring devices. Table 1, summarizes the well-established 2D materials that serve as active components in wearable sensors for remote health monitoring.

**Table 1:** Summary of 2D materials-based wearable sensors for wireless health monitoring.

Type of sensor	Active material	Monitoring parameters	Monitoring mechanism	Wearable platform	Response time	Mobile interface	Ref.
Pressure	Graphene nanosheets	Artery pulse	Resistive	patch	2 ms	✓	^40^
Pressure	Ti_3_C_2_T_x_ NS	Muscle contraction, artery pulse	Resistive	patch	11 ms	✓	^41^
Pressure	Ti_3_C_2_T_x_/protein	Pulse, phonation and knee bending	Resistive	Patch	7 ms	✓	^19^
Pressure	Ti_3_C_2_T_x_	Respiration	Resistive	patch	150 ms	✓	^42^
Pressure	Ti_3_C_2_T_x_	Body movements, intelligent robot motion	Resistive	patch	130 ms	✓	^16^
Pressure	Ti_3_C_2_T_x_	Muscle contraction and heart rate	Capacitive	Film	84 ms	✓	^47^
Pressure	Ti_3_C_2_T_x_ /PPy NWs	Muscle contraction	Resistive	Film	90 ms	✓	^72^
Strain	Hydrogen exfoliated Gr	Finger bending	Resistive	Film	--	×	^54^
Strain	FePS3/rGO	Breathing rate	Resistive	Band	2.38 s	✓	^14^
Strain	Ti_3_C_2_T_x_ -PAA/PVA	Muscle fatigue	Resistive	Film	--	✓	^18^
Strain	Ti_3_C_2_T_x_/PANI fiber	Artery Pulse, phonation, frowning	Resistive	Film	0.6 s	✓	^51^
Strain	Ti_3_C_2_T_x_	Finger movements	Resistive	Film		✓	^55^
Strain & Temperature	Ti_3_C_2_T_x_/ EDOT	Wrist pulse, Vocal vibration, finger bending, and Temperature	Resistive	Patch	0.33 s	✓	^52^
Electrochemical	Graphene	Viral (SARS-CoV-2)	Amperometric	Patch	--	✓	^59^
Electrochemical	Laser-induced Gr	Circadian rhythms and stress	Amperometric	Patch	--	✓	^21^
Electrochemical	MWCNT/ Ti_3_C_2_T_x_	Ions levels	Potentiometric	Patch	2 s	✓	^20^
Electrochemical	Ti_3_C_2_T_x_/PB	Sweat glucose, lactate, and pH	Amperometric	Patch	--	✓	^13^
Optoelectronic	Graphene quantum dots	Heart rate, arterial oxygen levels, and respiratory rate	Optical	Bracelet	50µs	✓	^56^

In the case of sensing pressure and strain, exfoliated 2D materials have layered structures and few nanometer interlayer spaces giving excellent piezoelectrical capabilities with high gauge factors^33^. Therefore, sensors based on 2D materials exhibit a very high sensitivity and can detect even the weakest pressure and stress. However, the use of 2D materials in electrochemical sensors has a lot of benefits that eventually enhance the sensitivity of the sensor^11^. The selectivity of 2D materials is primarily affected by their excellent carrier mobility and huge surface area. Moreover, the active sites in 2D materials give them electrocatalytic capabilities that enhance the recorded current while lowering the redox potential when the analyte is present. Regarding temperature sensing, the batter electrical conductivity and carrier mobility of 2D materials enable the investigation of temperature variations through altering resistance^34^.

## Working principles and mechanisms of wearable sensors

**Fig. 2:**
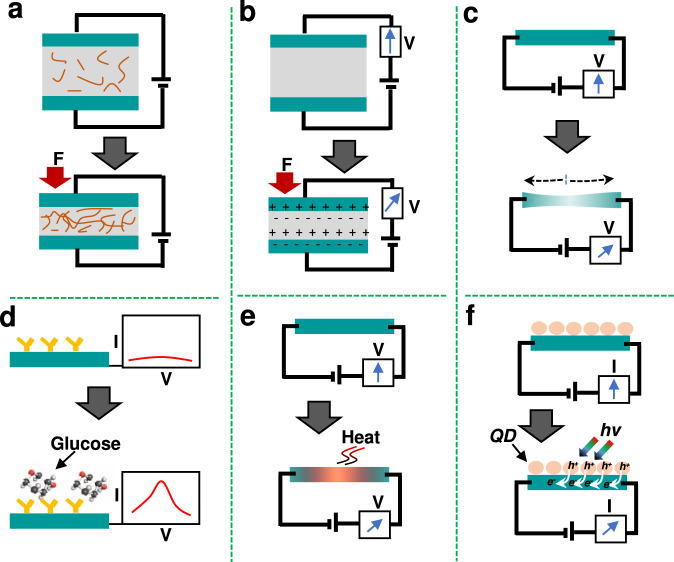
Schematic diagram of the mechanotransduction mechanisms in wearable sensors;. **a** piezoresistive pressure sensor, **b** piezo or triboelectric pressure sensors, **c** stress sensor, **d** electrochemical biosensor, **e** temperature sensor, and **f** optoelectronic sensor.

Wearable sensors and biosensors present an analytical tool that converts bio-signals into electrical or optical signals, *e.g*., physiological signals such as body temperature, heart rate, and motion that can be monitored and quantified in real-time. Wearable sensors are categorized based on their monitoring mechanism, *e.g*., pressure, strain, electrochemical, temperature, and optoelectrical sensors (Fig. 2**)**. Wearable pressure sensors can aid in the monitoring of human health conditions and enable huma-machine interface. Wearable pressure sensors based on 2D materials have opened up new avenues for monitoring and detecting human vital signs. For instance, a pressure sensor can monitor cardiovascular disease in terms of irregularities in blood pressure and heart rates. Due to their portability and wearability, a flexible pressure sensor can interface closely with human skin to continually convert tiny bio-signals into electric signals, including current, resistance, and voltage changes, by using triboelectric, piezoresistive, and piezoelectric mechanisms, respectively (Fig. 2a, b). Here, wearable pressure sensors are divided into four categories: piezoresistive, piezoelectric, triboelectric, and capacitive sensors^35^. The wearable piezoresistive sensor is used to track human movement signals by examining changes in resistivity and it has a long lifespan and rapid response time^36^, whereas wearable piezoelectric sensors monitor signals by electrostatic induction and piezoelectric effect^37^. Wearable capacitive sensors are used to sense motion based on changes in capacitance^38^. Typically, wearable triboelectric sensors consist of a triboelectric layer sandwiched between two parallel electrodes, which generate current or voltage signals upon pressure by triboelectric effect and electrostatic induction^37^.

The principal monitoring mechanism for wearable strain sensors (*e.g*., resistive- and capacitive-type sensors) is based on their change in dimension upon stretching (Fig. 2c). Owing to their comfortable interface with the human body, wearable strain sensors have attracted a lot of attention for human body motion and healthcare monitoring^39^. Furthermore, their mechanical elasticity and flexibility offer excellent skin compatibility, and they are sweat-resistant. Wearable biosensors, also known as electrochemical biosensors, are able to monitor biomarkers without being intrusive and represent a rapidly developing telehealth technology. Electrochemical biosensors measure biological binding activity through the change of current, resistance and capacitance of the biosensor surface (Fig. 2d). They enable the monitoring of bodily fluids such as saliva, interstitial fluid, tears, urine, and sweat, and are of specific interest as user-friendly and portable substitutes for existing analytical instruments in the healthcare field. These body fluids include numerous significant biomarkers, such as proteins (*e.g*., hormones, lactate dehydrogenase, and so on), tiny molecules (*e.g*., uric acid, glucose, amino acid, and cortisol), and ions (*e.g*., sodium, potassium, and calcium), which deliver clinically useful information for the treatment of numerous metabolic illnesses including cancer, diabetes, and periodontal diseases. For example, sweat biomarkers yield crucial perceptions regarding metabolic conditions (*e.g*., alcohol and glucose levels). Temperature is a critical marker of human health and sickness in both daily lives and in the context of healthcare. The majority temperature sensor measure temperature by changes in the electrical properties (e.g., current or resistance) of materials (Fig. 2e). In particular, skin-integrated wearable temperature sensors are often used to measure the basal temperature of newborns and infants. Additionally, real-time and continuous monitoring of body temperature may provide information to anticipate human illness by early fever detection.

The wearable optoelectronic sensor is a promising candidate for healthcare because it can monitor vital health signs precisely and in real time. The sensing mechanism of an optoelectronic sensor detects specific wavelength light into the skin and optically determines variation in blood vessel volume driven by the cardiac cycle (Fig. 2f). These types of wearable sensors enable the tracking of a variety of important signals, such as arterial oxygen saturation, heart rate, and respiratory rate.

## Applications of wearable sensors and biosensors

Nowadays, wearable sensors are becoming more prevalent, intelligent, and versatile for healthcare applications. The data obtained by wearable sensors can be of enormous importance to healthcare services and can be used to gain an understanding of a person’s health status. However, remote health monitoring systems based on wearable sensors and the Internet of Things (IoT) provide an efficient and affordable remedy that encourages the elderly (during pandemic situations) or people who have long-term illnesses (*e.g*., diabetes and cardiovascular disease) to stay in the comfort of their home instead of costly healthcare facilities. Also, these systems will allow doctors to monitor the vital physiological signs of patients in real-time, examine their health status, and give advice from a distance. This section discusses the use of wearable sensors and biosensors based on different 2D layered materials (*e.g*., graphene, rGO, Ti_3_C_2_T_x_, black phosphorus, FePS_3_, and WS_2_) for a remote health monitoring system.

### Wearable pressure sensors

Wearable pressure sensors have gained a lot of interest in the field of human health monitoring and human-machine interaction because they can continuosly monitor the physiological signals of the human body in real-time. Wearable pressure sensors have recently improved in terms of sensitivity and sensing range, but their thick active material films make them unsuitable as electronic skin (e-skin) and reduce their comfort as wearable sensors. The development of wearable pressure sensors that are tiny, flexible, highly sensitive, and reasonably priced is quite challenging. However, 2D materials are promising prospects for making compact (skin-integrated) and highly sensitive wearable pressure sensors due to their multi-layered structure with micro- and millimeter spaces and excellent conductivity.

**Fig. 3:**
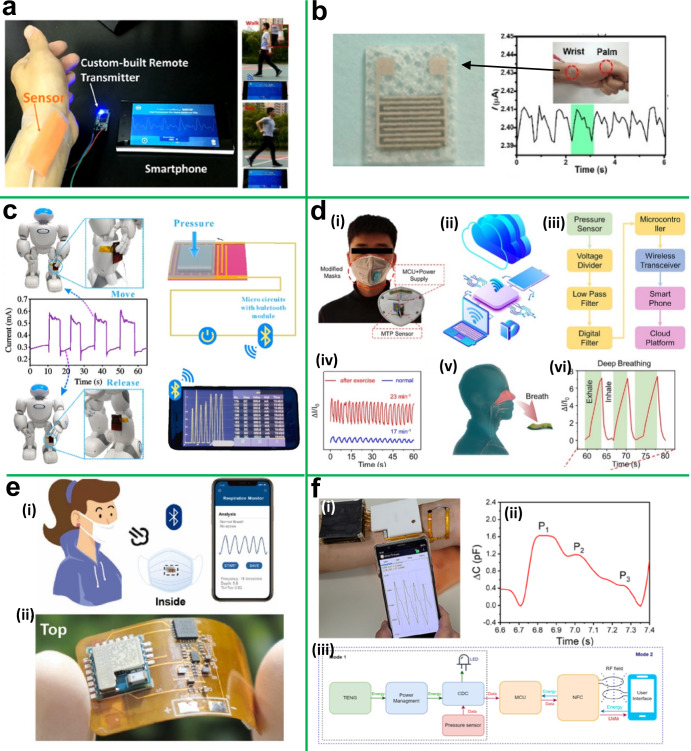
Wearable pressure sensors. **a** Image of graphene-based pressure sensor assembled on the wrist to remotely monitor pulse rate during walking and jogging. Reprinted with permission^40^. Copyright 2019, Elsevier. **b** Ti_3_C_2_-based pressure sensor for wireless wrist pulse monitoring. Reprinted with permission^41^. Copyright 2019, American Chemical Society. **c** Ti_3_C_2_-based pressure sensor to monitor movements of the robot and detected current signals transfer to the mobile device via Bluetooth communicator (Reprinted with permission^16^. Copyright 2020, American Chemical Society. **d** Smart face mask for wireless detecting opiate overdoses and monitoring respiration; (i) image of a smart mask with integrated Ti_3_C_2_ pressure sensor unit, power supply unit, and wireless data transmission unit, (ii, iii) a schematic and block diagram of wireless respiration monitoring system, (iv) respiration cycle monitoring before and after activity, (v) diagrammatic representation of human exhaled air detection, (vi) monitoring deep breathing cycle. Reprinted with permission^42^. Copyright 2021, American Chemical Society. **e** MWCNT@Ti_3_C_2_ fabric-based smart mask for respiration monitoring; (i) schematic view of an integrated mask with detection tag and wireless data transmission and (ii) front and back side images of respiratory detection tag. Reprinted with permission^43^. Copyright 2022, Elsevier. **f** Wearable Ti_3_C_2_-based remote health monitoring system; (i) photograph of wearable health monitoring system assembled on the wrist and recorded pulse transferred to a smartphone, (ii) zoom view for single wrist pulse, and (iii) system block diagram for remote health monitoring. Reprinted with permission^47^. Copyright 2022, Elsevier.

Figure 3 displays some wearable pressure sensors based on 2D materials for monitoring the physiological signals and motions of the human body remotely and in real time. Chen et al. developed graphene film by using a simple self-assembly method and incorporated a pressure sensor by applying films on micropatterned polydimethylsiloxane substrate^40^. The linearity and conductivity of the pressure sensor could be perfectly balanced by controlling the structure of the graphene films. The obtained pressure sensor with a thin and highly conductive graphene layer has a sensing range of 20 kPa and high sensitivity of 1875.5 kPa^−1^, a small response time (0.5 ms), and high stability (15000 cycles), which can be turned into a wearable prototype device for the health monitoring device. Afterward, they developed a wireless wearable pulse monitoring system (Fig. 3a) that can precisely identify and monitor very faint wrist artery pulse signals in real time when the wearer is walking or running. Ying et al. reported a highly flexible MXene (Ti_3_C_2_) nanosheet-based wearable pressure sensor for human motion detection^41^. The fabricated pressure sensor had a superior detection limit (10.2 Pa), excellent reproducibility, quick response (11 ms), and low power consumption (10^−8 ^W). Further, Ti_3_C_2_-based pressure sensor was affixed to human skin to detect various physiological signals ranging from large movements to subtle deformations. As shown in Fig. 3b, the human wrist-mounted Ti_3_C_2_-based pressure sensor was able to detect radial artery pulse signals (80 beats per minute) with regular waveforms. To monitor various touch signals, they developed an artificial e-skin using a Ti_3_C_2_-based pressure sensor array and coupled it to a wireless transmitter to enable wireless sensing of the human-machine interface. To construct a comfortable and robust pressure sensor, the same research group developed a breathable and highly sensitive Ti_3_C_2_@protein-based wearable pressure sensor on a piece of silk fabric for real-time monitoring of human physical activity^19^. The Ti_3_C_2_-coated silk fabric has a porous 3D network that offers high surface area, enough roughness and flexibility, and realizes excellent sensing performance with a quick response/recovery time of 716 ms, high sensitivity of 298.4 kPa^−1^, and a wide detection range up to 39.28 kPa. It also exhibits excellent sensing capability in monitoring human psychological signals (e.g., pulse, phonation, and knee bending) and functions as an artificial skin for real-time wireless biomonitoring and pressure distribution visualization.

Figure 3c depicts a pressure-sensitive microstructure inspired by human skin and developed using Ti_3_C_2_ and abrasive paper as a template. The surface morphology with randomly distributed bionic spinous microstructures increases the contact area of the conductive channels and enhances performance^16^. The optimized pressure sensor exhibits a low detection limitation of 4.4 Pa and high sensitivity of 151.4 kPa^−1^. This pressure sensor can be fastened to the wrist and fingers to measure artery pulse rate and bending movement, respectively, or affixed to the throat to monitor swallowing activity. Moreover, its microstructure provides a new way to improve wearable pressure sensor sensitivity and broaden the scope of monitoring human motion. The pressure sensor is attached to a robot’s surface to mimic the way skin senses the motion of the robot and wirelessly transfers detected current signals to the mobile device.

A new type of wearable pressure sensor based on Ti_3_C_2_@tissue paper was reported by Ying et al.^42^. The highly porous three-dimensional structure of the Ti_3_C_2_ coated tissue paper and high surface area of Ti_3_C_2_, shows ultrahigh sensitivity and wide detection ranges. The fabricated pressure sensor was able to measure human physiological signals, such as blood pressure, radial artery pulse, phonation, and different motion states (*e.g*., jumping, standing, and walking), and its wireless array was further used to monitor respiration and detect opioid overdose (Fig. 3d). Recently, Han et al. developed a smart mask based on Ti_3_C_2_@multi-walled carbon nanotube with an integrated wireless data transmission system for real-time respiratory monitoring (Fig. 3e)^43^. Interestingly, this pressure sensor able to provide 265% response at 90% humidity and batter response stability upon various mechanical deformation. The proposed sensor demonstrated remarkable respiratory sensing and precise recognition of varied respiration patterns. This study provides an excellent illustration of how a simple modification method can be used to enhance sensing capabilities upon deformation and shows promise for real time respiration monitoring. Furthermore, our research team has developed a BP-based piezoresistive pressure sensor for the human–machine communication interface^44^. BP was coated on fabric using aniline monomers in situ polymerization. The achieved BP@PANI-based sensor exhibits sensitivity in a wide range of pressure and has reasonable response time and excellent hysteresis in load–unload pressure.

The fabricated prototype auditory feedback system made of a six-pixel BP@PANI-based pressure sensor array that matches with a braille character set enables blind users to hear auditory feedback in response to touched braille text. This proposed system is very useful for people who are visually impaired or have difficulty speaking. To the best of our knowledge, the BP-based pressure sensor has never been employed in auditory feedback (touch to audio) before. Finally, we demonstrated a typical conversation between humans and machines, with potential for a smart braille audiobook. This research is critical in addressing the social and public issue of information exchange for people who have speech or vision problems. Despite significant advancements in skin comfort, robustness, and performance, a portable power supply remains a key obstacle to wearable pressure sensors. Many existing reports on flexible and wearable energy storage devices have shown that they are unable to consistently supply power to wearable sensors for real-time health monitoring. Therefore, our research team has developed a wearable biomonitoring system by integrating a Ti_3_C_2_/WS_2_-based supercapacitor and pressure sensor, which function as a portable power source and real-time health monitoring device, respectively^45^. This wearable system can track radial and carotid pulses as well as foot alignment during exercise. In addition, we recently reported black phosphorous (BP) based portable and wearable energy storage devices for power wearable pressure sensors^46^. We were able to effectively show the ability to wirelessly transfer real-time collected bio-signals to a mobile phone.

Recently, Esfandyarpour et al. developed a self-powered wearable pressure sensing system based on Ti_3_C_2_ for continuous real-time monitoring of human physiological signals^47^. As shown in Fig. 3f, the Ti_3_C_2_-based self-powered wearable pressure sensing system consists of Ti_3_C_2_-based triboelectric and pressure sensors. This system demonstrated an output power (TENG) of 816 mWm^−1^, sensitivity of 6.03 kPa^−1^, fast response of 80 ms, and a low detection limit of 9 Pa. The self-powered system primarily used two ways to monitor human health. First, a Ti_3_C_2_-based triboelectric sensor supplies power to the entire system continuously and in real time monitors wrist artery pulse. Second, the Ti_3_C_2_-based triboelectric sensor continues to power the Ti_3_C_2_-based pressure sensor and capacitance-to-digital-converter (CDC) while a mobile device wirelessly powers the microcontroller unit (MCU) and allows wireless power and data transmission. It is noteworthy that this proposed wearable health monitoring system is entirely driven by human motion, indicating a tremendous potential for commercialization. Overall, above mentioned wearable pressure sensors provide more detailed and trustworthy information, which opens the door to the commercial viability of wearable sensors.

### Wearable strain sensors

**Fig. 4:**
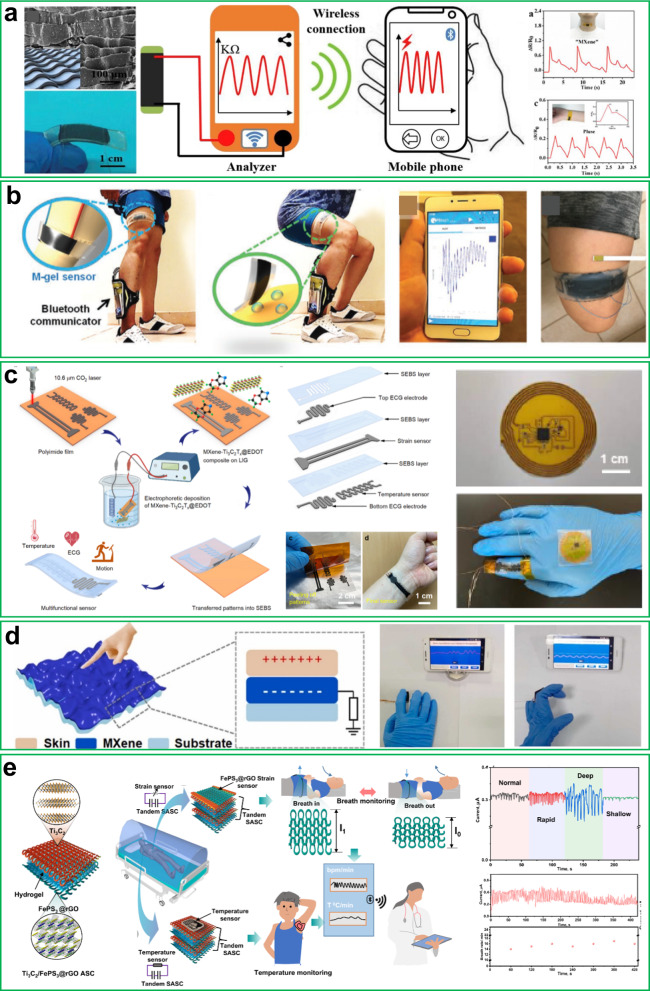
Wearable strain sensor. **a** Polyaniline@Ti_3_C_2_-based flexible strain sensor with a tile-like stacked structure for wireless detection of artery pulse and phonation. Reprinted with permission^51^. Copyright 2020, Elsevier. **b** Wearable wristband based on a Ti_3_C_2_ hydrogel strain sensor and Bluetooth module wrapped around a human thigh during a workout to measure the degree of muscle tiredness. Reprinted with permission^18^. Copyright 2021, Wiley. **c** Ti_3_C_2_@3,4-ethylenedioxythiophene/graphene composite-based wireless multifunctional sensors for strain, temperature, and heartbeat monitoring. Digital images represent the wearability and skin comfort of sensing system. Reprinted with permission^52^. Copyright 2022, Springer Nature. **d** Schematic illustration of the crumpled Ti_3_C_2_ film and the self-powered real-time monitoring system that wirelessly transmits varied degrees of finger bending and pressing signals to the smartphone. Reprinted with permission^55^. Copyright 2022, Elsevier. **e** Remote health monitoring system based on an integrated FePS_3_/rGO-based strain sensor and a flexible supercapacitor for real-time breathing rate and body temperature monitoring. Reprinted with permission^14^. Copyright 2022, Springer Nature.

Numerous fitness-related illnesses are on the rise around the globe and the elderly population grows daily. Therefore, it is crucial to monitor health conditions continuously. The increasing need to keep track of various physiological factors has prompted the development of wearable strain sensors, which represents an important advancement in telehealth. A wearable strain sensor could be a crucial tool to monitor activities and boost digital health technology. A variety of materials, such as polymers (polyaniline and polyvinylidene fluoride), carbon nanotubes, and silicon nanowires have been utilized to fabricate wearable strain sensors^48–50^. For now, 2D layered materials (*e.g*., Ti_3_C_2_, graphene, *etc*.) can precisely detect both large human motion activities (like bending a leg or finger joints) and tiny movements (such as speaking and breathing), and all of these motions can be detected remotely. Figure 4 illustrates a few wearable strain sensors based on 2D materials for remote health monitors.

Taking inspiration from overlapped rooftop tiles, Chao et al. introduced stacked hierarchical microstructure to piezoresistive film *via* the composition of Ti_3_C_2_ nanosheet and polyaniline fiber^51^. Overlap of Ti_3_C_2_ and polyaniline fibers provide additional conductive pathways to maintain the continuity of the conductive path. Advantage from the tile-like stacking structure of composite, the strain sensor features a sliding and crack extension sensing mechanism upon stress process, which allow for high sensitivity over a broad strain range in sensor. As a result, the strain sensor reported high sensitivity with a gauge factor of 2369.1, an ultralow detection limit of 0.153% strain, and good reproducibility. As a proof-of-concept demonstration, the wearable strain sensor was integrated into a wireless transmitter for wirelessly monitoring human activity including bending fingers and elbow joints as well as detecting sound and human wrist pulse (Fig. 4a).

In 2021, Alshareef et al. reported Ti_3_C_2_-polyacrylic acid@ polyvinyl alcohol composite hydrogel that they attached to a wearable wristband sensor^18^. This wearable hydrogel sensor changes resistance in response to strain and pH level. Based on the pH of sweat, the cation selectivity of the Ti_3_C_2_ surface may become either strong or weak. A wearable Ti_3_C_2_ hydrogel sensor was attached to the skin during exercise and was able to determine the pH levels in the human sweat based on the electrochemical response of the hydrogel. Additionally, a strain sensor can be integrated with a Bluetooth communicator to transfer recorded electrochemical responses directly to a mobile phone (Fig. 4b). They were successful to detect a pH between 4 to 6. This sensor presents a creative idea for developing high-performance wearable strain sensors for remote medical care.

Figure 4c depicts a Ti_3_C_2_/laser-induced graphene@3,4-ethylenedioxythiophene (EDOT)-based multifunctional wearable strain sensor for simultaneous monitoring of temperature, strain, and electrocardiogram^52^. The Ti_3_C_2_/graphene@EDOT composite showed a good temperature coefficient of resistance (0.86%), high strain sensitivity, and lower skin contact impedance, making it a promising wearable sensor for monitoring diverse biosignals. The researchers wirelessly monitored in real time various human-induced subtle and large deformations such as vibration of the Adam’s apple, arterial pulse, and large body joint bending motions. Little research has been done on wearable strain sensors based on graphene and black phosphorus (BP) for remote health monitoring. For instance, a BP-coated cellulose paper-based strain sensor was fabricated and used for human motion monitoring^53^. The BP strain sensor was attached to the human neck, wrist, and hand for real-time monitoring of human motion. The demonstration of BP on cellulose paper using a low-cost method offers up new possibilities for wearable sensors but remotely monitoring health is still challenging. Further, a waterproof flexible wearable strain sensor was developed using poly(vinylidene difluoride) with polymer-functionalized graphene^54^. The researchers successfully demonstrated wireless control of a robotic arm through this graphene-based strain sensor, indicating future potential applications for human activity monitoring, bio-functional prosthetic limbs, and healthcare.

The majority of physiological signals recorded previously have been based on piezoresistive strain sensors, whereas only a few reports have used piezoelectric or triboelectric strain sensors for remote health monitoring. Also, a wearable strain sensor requires a portable power supply to function. Hence, Su et al. developed self-powered wearable strain and pressure sensors based on a triboelectric nanogenerator (TENG) by fabricating crumpled Ti_3_C_2_ film on a stretchy balloon, which greatly increased the mechanical strength and interfacial adhesion on human skin (Fig. 4d)^55^. A sensor based on TENG possesses excellent tensile properties (such as a linear tension ratio of 400% and maximum areal strain ratio of 2150%) and sensitivity (2.35 V kPa^−1^), which indicate a great potential for detecting human movements. Figure 4d depicts a wireless real-time motion-detecting system comprising three components: Ti_3_C_2_-based pressure/strain sensors, data processing and transmission, and data receiving and evaluation. The Ti_3_C_2_-based sensor serves as the sensing unit to identify the strain or pressure signals and convert them into electrical signals. Then, the electric signals are evaluated and relayed *via* the Bluetooth module, where they can then be wirelessly transferred to a mobile phone. A wearable strain sensor based on FePS_3_ (metal phosphorous chalcogenides) and portable energy power storage device (Ti_3_C_2_/FePS_3_-based asymmetric supercapacitor) was reported by Pumera et al.^14^. A developed all-in-one textile-based breathing monitoring band (comprising a wearable strain sensor and power supply device) was wrapped to the abdomen to continuously track a person’s breathing patterns and wirelessly transmit data to a mobile device (Fig. 4e). Further, they prepared a smart portable temperature sensor patch integrated with the Ti_3_C_2_/FePS_3_-based portable power source. The sensor was attached to a person’s armpit to track their body temperature in real time.

### Wearable optoelectronic sensors

**Fig. 5:**
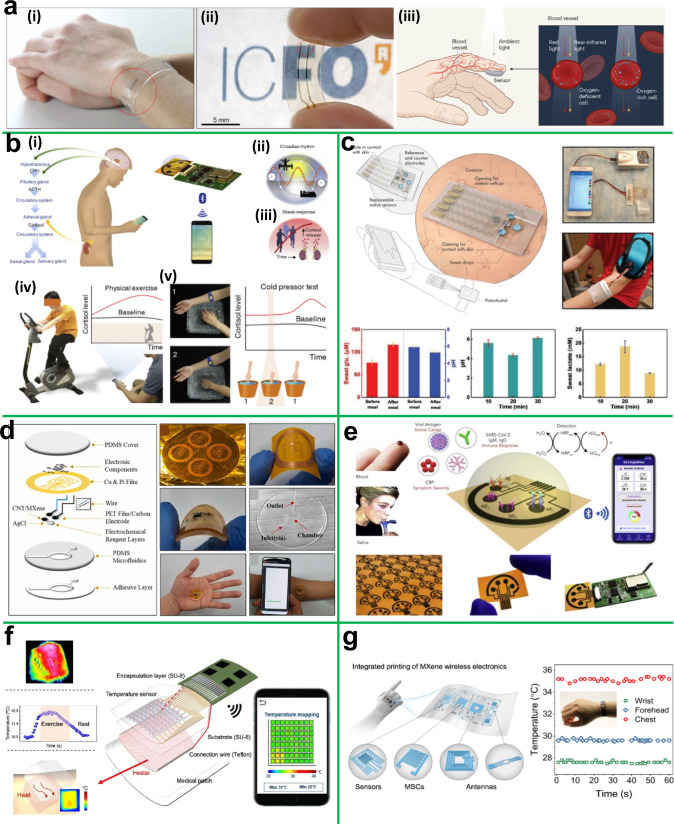
Wearable optoelectronic, electrochemical, and temperature sensors/biosensors. **a** Graphene QD-based photodetector; (i, ii) digital image of a flexible, wearable, and transparent photodetector and (iii) schematic illustration for detecting artery blood flow and heart rate. Reprinted with permission^56^. Copyright 2019, The Authors, some rights reserved; exclusive licensee American Association for the Advancement of Science. Distributed under a Creative Commons Attribution-NonCommercial License 4.0 (CC BY-NC) http://creativecommons.org/licenses/by-nc/4.0/. **b** Graphene-based bio-sensing system for wireless stress hormone monitoring (i) schematic illustration of a stress sensing system for monitoring cortisol levels from sweat and saliva, (ii, iii) conceptual representation of cortisol changes controlled by circadian rhythm and driven by physical and emotional stress, (iv) schematic illustration of stress monitoring and data wirelessly transmitted to a smartphone, and (v) stress monitoring in relation to temperature. Reprinted with permission^21^. Copyright 2020, Elsevier. **c** Prussian Blue@Ti_3_C_2_-based wearable biosensing patch for remote determination of sweat biomarkers such as lactate, pH level, and glucose. Reprinted with permission^58^. Copyright 2019, Wiley. **d** Wireless and battery-free circular biosensor patch for monitoring potassium ions in sweat and wirelessly transmitted to a smartphone. Reprinted with permission^20^. Copyright 2021, Elsevier. **e** Telehealth platform for the diagnosis of SARS-CoV-2 in human blood and saliva using a graphene-based electrochemical biosensor. Reprinted with permission^59^. Copyright 2020, Elsevier. **f** Wearable and wireless graphene heater for temperature tracking and thermal therapy on a vast region of skin. Reprinted with permission^17^. Copyright 2022, The Authors, some rights reserved; exclusive licensee American Association for the Advancement of Science. Distributed under a Creative Commons Attribution-NonCommercial License 4.0 (CC BY-NC) http://creativecommons.org/licenses/by-nc/4.0/. **g** Wearable Ti_3_C_2_-based temperature tag wirelessly measure human wrist, forehead and chest temperature. Reprinted with permission^60^. Copyright 2022, Springer Nature.

In telehealth, one of the most frequently utilized optical imaging techniques is photoplethysmography, which is employed to monitor physiological parameters, including cardiac output, oxygen saturation, respiration, and blood oxygen. In short, this technique can detect volume changes over the entire human body and can estimate how much light is absorbed and reflected by blood arteries in living cells. As shown in Fig. 5a, a graphene quantum dot-based wearable optoelectronic sensor can continuously and accurately monitor a variety of human vital signs^56,57^. The sensor has strong response tunability (up to 10^5 ^A W^−1^), excellent low light sensitivity (down to ~pW), and a fast response time of 50 µs. This proposed sensor can monitor blood pulse oxygenation, heart rate, and respiration as well as ultraviolet light exposure. While the optoelectronic sensor monitors the various parameters, the data are displayed and saved on a smartphone interface coupled to the wearable sensor through Bluetooth. Moreover, this wearable optoelectronic sensor can run without a battery because the sensor is wirelessly charged by a smartphone.

### Wearable electrochemical biosensors

This section focuses on the application of 2D layered materials-based wearable electrochemical biosensors for remotely identifying illness biomarkers. A wearable sweat analyzer could be the next development in the non-invasive detection of health biomarkers. An interesting graphene-based smart and wireless health monitoring system was reported by Gao et al. for the investigation of cortisol in human sweat (Fig. 5b)^21^. The microfluidic biosensor patch used in this study has remarkable mechanical flexibility and skin comfort. Graphene-based biosensors exhibit significantly better sensitivity (3.72 nA mm^−2^) compared to conventional electrodes, such as glass carbon electrode (0.68 nA mm^−2^) and screen-printed electrode (2.41 nA mm^−2^). To improve the accuracy of the stress sensing system was designed by three working electrodes with reference and counter electrodes. This design approach can be used as a hormone panel sensor for the detection of various stress-related hormones. To establish a compact wireless stress monitoring system, a graphene sensor patch was integrated with a signal processing unit, power supply unit, and Bluetooth module. As a result, this established remote healthcare system presents precise results and a good correlation between hormones and the dynamic stress-response profile. The proposed approach might be used to advance technologies in health management and individualized personal performance.

Figure 5c represents a multifunctional wearable electrochemical biosensor based on Ti_3_C_2_@Prussian Blue composite for the detection of different biomarkers in sweat^58^. The unique design of the electrochemical biosensor as a tri-phase (solid-liquid–air) interface, improves the sensitivity and durability of in vitro experiments. This biosensor was made to detect three key biomarkers in sweat: lactate, glucose, and pH. The biosensor sensitivity for measuring glucose and lactate levels in artificial sweat was 35.3 µA per mMcm^2^ and 11.4 µA per mMcm^2^, respectively, and also monitoring well in on-site sweat monitoring while exercising. Additionally, they performed a real-time sweat test by attaching a wearable patch to the wrist and monitoring changes in lactate, glucose, and pH during intense exercise before and after meals. Finally, this wearable patch wirelessly transmitted real-time data to a smartphone for analysis.

Park et al. reported a unique design of Ti_3_C_2_@multiwalled carbon nanotube-based wearable electrochemical biosensor patch that is small, lightweight, battery-free, and capable of monitoring potassium ion concentration in sweat (Fig. 5d)^20^. For real-time and remote monitoring of potassium ions, a wearable biosensor patch was integrated with a microfluidic sweat harvesting device, and near-field communication (NFC) was utilized to transfer measured signals to a mobile phone wirelessly. This electrochemical biosensor patch was built with a 3D-printed microfluidic channel to capture more sweat and minimize sensor surface contamination. It was found that biosensor patch sensitivity is 63 mV dec^−1^, which can be boosted to 173 mV dec^−1^ *via* NFC with a potassium (K^+^) concentration range of 1-32 mM. With the development of MXenes, research on Ti_3_C_2_-based wearable electrochemical biosensors for telehealth is also progressing. However, wearable biosensors on Ti_3_C_2_ are still in the early stage of development when compared to other 2D materials like graphene, with very few biosensors and remote healthcare applications. The present COVID-19 pandemic has caused a global disaster, with around 5.42 million people dead worldwide (from December 2019 to 2021). Even though vaccination campaigns are in full force, the new variants are still causing problems; therefore, a highly sensitive and reliable early SARS-CoV-2 infection detection biosensor is still required to reduce transmission both within and between communities. For quick detection of COVID-19 biomarkers, Torrente-Rodrigues et al. developed an ultrasensitive, low cost, and wireless electrochemical platform called SARS-CoV-2 RapidPlex (Fig. 5e)^59^. This electrochemical biosensor is made using laser-engraved graphene, which allows for quick detection of various COVID-19 biomarkers (*e.g*., anti-spike protein IgG and IgM, nucleocapsid protein, and C-reactive protein) and provides important information regarding immune response, viral infection, and disease severity. As stated above, existing power sources for powering wearable sensors and biosensors are not biocompatible because the sensors are likely to come into contact with many body parts. However, the power source device must be biocompatible in order to prevent unwanted immune responses and other deleterious effects. Concerning this, Prof. Pumera’s research team created a sweat rechargeable energy storage patch made of Ti_3_C_2_/polypyrrole-carboxymethylcellulose composition^7^. They successfully measured blood glucose by powering a glucose meter and demonstrated that a sweat-chargeable patch can power a variety of biosensors that only require power for a short period of time. Expanding on this concept, if such type of bio fluid-based energy storage device integrates with existing or upcoming biosensors that may monitor various biomarkers in real time and remotely.

### Wearable temperature sensors

Human body temperature is a crucial parameter for the noninvasive prognosis of various illnesses and physical conditions. For instance, an armpit temperature of 37.2 °C or higher is a sign of fever. Therefore, it is crucial to assess body temperature accurately and precisely. Recently, a multifunctional wearable thermal sensor patch was developed by M. Kang and coworkers^17^. The patch is made of a graphene-based pad and capacitive sensor that is connected with a readout integrated circuit for analyzing and relaying the measured data to a smartphone (Fig. 5f). This wearable sensor patch can continuously and in real-time monitor temperature during exercise and assist in detecting health issues by acquiring the temperature distribution profile of a specified area. Moreover, a graphene heater placed beneath the wearable temperature sensor may enhance the healing rate of wounded skin, probably by vasodilation. Shao et al. developed additive-free Ti_3_C_2_ ink and fabricated a wearable integrated system for wireless communication, energy harvesting, and temperature sensing^60^. Fig. 5g depicts the operation of the Ti_3_C_2_ radio frequency identification (RFID) temperature monitoring system, which is based on the RFID backscatter coupling between the temperature tag and reader. They successfully monitor human body temperature and transfer the recorded data to a mobile device. Little progress has been made in wearable wireless temperature sensors using 2D materials. However, there is still scope to use other 2D materials, such as TMDs family containing SnSe, Bi_2_Te_3_, Bi_2_Se_3_, etc.^61^, all with excellent thermoelectric properties.

## Limitations and Challenges

**Fig. 6:**
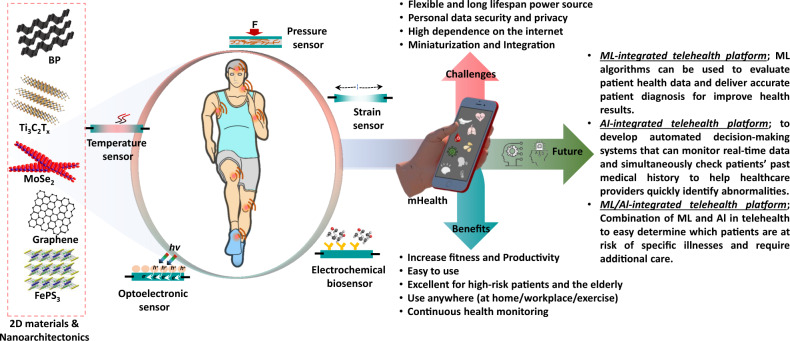
Highlights future outlook. 2D materials and nanoarchitectronics-based wearable sensors for telehealth applications, benefits, challenges, and future outlooks.

To investigate the most recent advancement trends of wearable sensors for wirelessly monitoring vital signs, we summarized and examined a list of 2D materials-based wearable sensors. In recent years, owing to the tremendous growth of 2D materials such as graphene, MXenes and TMDs families, they have found widespread application in a variety of wearable sensors, including a pressure sensor, electrochemical sensor, triboelectric sensor, temperature sensor, strain sensor, optoelectronic sensor, and so forth. Furthermore, some researchers have integrated multifunctional wearable sensors all in one substrate for simultaneous physiological signals acquisition^17,52^. However, those multifunctional sensors concentrate on the integration of mechanical sensors (*i.e*., pressure and strain sensors) with the temperature sensor. Therefore, to establish a more multifunctional remote health monitoring system, mechanical and electrochemical wearable sensors would be a potential path for comprehensive body condition assessment. Although physiological signals monitored wirelessly have been proved in many reports, there is still a long way to go from laboratory- to large- or industrial-scale. Figure 6 depicts the current achievements, challenges, and future of a 2D materials-based wearable sensor for telehealth applications.

First, mass production of all types of 2D materials is quite challenging and costly. Presently, graphene and GO can be synthesized on a large scale; however, the difficulty of other 2D materials (MXenes and TMDs families) to be made on such a massive scale limits their future industrial application. Moreover, environmental stability is a barrier for 2D materials-based wearable sensors in biomedical applications. The interplay of 2D materials with biological environments (*e.g*., sweat and saliva) might impair wearable sensor performance. Some 2D materials applications are restricted by environmental instability, which must be addressed without impairing the sensitivity of the sensors. For instance, BP is unstable at standard atmosphere but its surface can be modified through polymer functionalization or doping elements, which can significantly enhance its stability. Also, there are many unexplored 2D materials available (*e.g*., V_3_C_2_T_x_, hexagonal boron nitride, NiPS_3_, and so on) that could be used for wearable sensors and human-machine interfaces.

Second, because wearable sensors utilized in health monitoring and human-machine interaction are attached directly to the skin or injected into the body, there are concerns regarding human safety and comfort, while wearing them. For instance, the wearable sensor containing a used Li-ion battery could be hazardous if mistreated or of poor quality. Because wearable sensors integrated with these power sources are always close to the human body there is a huge safety risk. Apart from this, a portable power source with excellent mechanical stability and longevity is also required, which will improve both the wearing comfort and sensor architecture.

Third, the major constraint is personal data privacy. Wearable sensors acquire personal information and link to the internet, so it is important to develop high-security features to ensure patient personal data is not exposed or hacked. Fourth, it is challenging to integrate many components (power source, data transmitter, or antenna) with reasonable signal strength in a tiny space. Therefore, close collaboration with industry or electrical engineers is required to encourage the signal process and transmission unit with a flexible, lightweight, compact size, and low power.

Finally, the most significant aspect of RHMS is the interpretation and validation of acquired health data using the big data available to healthcare systems. The future of digital health anticipates the merging of artificial intelligence- (Al) and machine learning (ML)-based investigations with a wearable health monitoring system. Successful integration will allow for patient-oriented serious illness management (*e.g*., diabetes, chronic, cardiovascular disease, and hypertension) reduce clinical visit frequency, and offer a personalized on-demand diagnosis. Moreover, the numerous human signals monitored by wearable sensors should be evaluated by the appropriate medical specialists and more meaningful information can be interpreted by comparing the results of standard medical testing.

## Discussion

Wearable sensors, as the primary device for sensing human vital signals, are fundamental components of flexible electronics and serve critical roles in a variety of sectors, including wearable health monitoring and human-machine interaction. In this review, we have given an outline of wearable sensors/biosensors and proof-of-concept remote healthcare systems based on 2D materials that have been developed in last four years. Emerging 2D materials and nanoarchitectonics-based wearable sensors offer many advantages to users, from monitoring vital signs like body temperature, arterial oxygen saturation, sweat biomarkers, breathing cycle, heart rate, and movements as well as early viral/flu identification (*i.e*., SARS-CoV-2). We provided an overview of different wearable sensors (pressure, strain, electrochemical, optoelectronic, temperature sensors), including how they operate, their detection mechanism, integration with electronic circuits, and application in human body monitoring.

First, we described an integrated healthcare system using pressure sensors to deliver real-time information on human health. This system mainly reported monitoring heart rate (attached to human skin) and breathing cycles (a mount-on face mask). Here described wearable pressure sensors primarily function under repeated loading and unloading in typical ambient circumstances. Nonetheless, in practical applications, the pressure sensor will come into contact with challenging environmental conditions, including sweating and high humidity, which may affect the stability of 2D materials (e.g., MXene) and the performance of sensors. Therefore, more research into the mechanism behind working stability in artificially complex environment situations is necessary^62,63^. Additionally, effective packing procedures with little impact on performance and nano/microstructures of 2D materials should be investigated.

Next, we focused on tracking human activities via integrated wearable stress sensors, which can quickly identify both large and tiny movements of a person and transmit data to healthcare providers or user’s smartphone. This involved a range of movement monitoring such as heartbeat, muscle fatigue, body joint bending, a vibration of Adam’s apple, and breathing rate. The use of all-in-one textile-based stress sensors for remote health monitoring was also discussed. Excellent stretchability and sensitivity were displayed by resistive type strain sensors; nevertheless, they suffer from hysteresis, poor linearity in the outer range of strain, and instability because of environmental changes (humidity and temperature variations). For instance, MXene can be oxidized in the open environment, which reduces the shelf-life of the sensors and causes problems with consistency, reliability, and repeatability. The use of anti-oxidants and a modified MAX phase synthesis process have shown promise in addressing the stability issue with MXenes, but this work is still being done on a lab scale^64,65^. Moreover, there are also difficulties in the creation of strain sensors that can detect dissociated strain in all directions and multiple planes of deformation. To get over this restriction, more research requires on innovative sensor architectures and three-dimensional nanostructures.

Then we described electrochemical biosensors to remotely monitor various biomarkers such as stress hormones, lactate glucose, sweat pH, and COVID-19 in saliva. The majority of biosensors only assess a few biomarkers. In the future, more research should be required to develop new 2D materials-based sensors and expand the understanding of sensing to monitor a variety of biomarkers. Expanding the use of wearable sensors in the healthcare sector will require an understanding of biofluid composition and its relationship to health and specific disease conditions. We also provided an overview of 2D materials-based wearable optoelectronic and temperature sensors for telehealth applications. Graphene has been the most extensively researched 2D material for temperature and optoelectronic sensors, and it has progressed from research to clinical use. However, other 2D materials, such as TMDs, black phosphorous, and MXenes are quickly filling the gap. In contrast to graphene, these materials have properties like bandgap range and photocurrent generation capability making them potential candidates for the development of the future wearable optoelectronic sensor.

We anticipate that wearable sensors based on 2D materials will emerge as a new sensing platform for the healthcare industry. There are still significant challenges remaining to show that 2D materials-based wearable sensors and biosensors can beat commercially available sensors on all critical features. Apart from the price of a wearable sensor, some of the challenges, such as the necessity for a flexible power source (or self-power), 2D materials stability and mass production, health monitoring data privacy, and so on, must be thoroughly investigated. The majority of works have focused on using graphene oxide, Ti_3_C_2_T_x_, and BP; however, there are still many viable 2D materials that require investigation.

Furthermore, RHMS enabled by wearable sensors/biosensors and large data processing has enormous promise for providing users with real-time health monitoring information and diagnostic tools. Future studies on improving the sensing accuracy of 2D materials-based wearable sensors could broaden their applicability. Following, we suggest encouraging future study and application in the area of wearable sensors and telehealth; (i) low-cost industry scale fabrication techniques must be developed. Due to the complexity of the existing synthesis process for 2D materials, their future industrial application is prohibited. However, Y. Gogotsi et al. and X. Feng et al. have reported industry-scale manufacturing methods of synthesis MXene^66,67^. (ii) To build automated artificial intelligence (Al)-based decision-making systems (Al integrate with RHMS) that monitor real-time data and simultaneously check patients’ past medical history to help healthcare providers quickly identify abnormalities^68^. (iii) To create more advanced multi-parameter sensing wearable devices and telehealth platforms with robust functionality and inexpensive cost that may be used in a variety of health care^69^. (iv) health signal processing and data transmittance units with a flexible circuit board, compact sizes, low power use, and lightweight need to develop by close collaboration with the electrical engineer^70^. The existing circuit was fabricated using conventional chips, which are inappropriate for specific applications, like the chip placed in a contact lens for measuring intraocular pressure^71^. It is essential to develop a small size circuit system with a long operation life to keep track of human physiological information. We believe that this review gives readers a comprehensive overview of the current situation, challenges and future outlook of innovative wearable sensors based on 2D materials for remote healthcare systems.

## Data Availability

The data that support the findings of this study are available within the paper. Moreover, sources of all the figures are provided in the paper.
